# New hope for a microRNA therapy for pulmonary arterial hypertension

**DOI:** 10.3389/fgene.2013.00137

**Published:** 2013-07-19

**Authors:** Jyoti Mehta, Prasanna T. Parthasarathy, Richard Lockey, Narasaiah Kolliputi

**Affiliations:** Department of Internal Medicine, Division of Allergy and Immunology, Morsani College of Medicine, University of South FloridaTampa, FL, USA

Pulmonary arterial hypertension (PAH) is a progressive disease characterized by pulmonary vascular remodeling and right heart failure. In its advanced stage, PAH demonstrates muscularization of distal pulmonary arterioles, concentric thickening, and obstruction of vascular lumen due to the proliferation of pulmonary artery endothelial cells (PAECs) and pulmonary artery smooth muscle cells (PASMCs) (Steiner et al., 2009). Ultimately, such arteriopathy in PAH leads to a life threatening increase in resistance of PAECs and PASMCs to apoptosis. However, the molecular and cellular basis for vascular remodeling in PAH has not been elucidated. This pathogenesis of PAH shares similarities with cancer, in which angiogenesis is characterized by an increase in proliferating endothelial cells and atypical morphology of tumor vasculature (Hoff and Machado, 2012). Several studies suggest that micro-RNAs (miRNAs) play vital roles in cancerous cells. The miRNA 7–19 cluster located on chromosome 13 consists of 7 miRNAs and was among the first miRNA clusters described and upregulated in lung cancer (Billeter et al., 2012). Additionally, miR-206s regulates proliferation, apoptosis and differentiation of PASMCs (Jalali et al., 2012). miR-16's overexpression in alveolar epithelial (A549) cells downregulates serotonin transporter (SERT) and upregulates epithelial sodium channel, EnaCβ, in the presence of serotonin (Parthasarathy et al., 2012). Given an increased understanding of miRNAs' roles in cancer and the similarities between angiogenesis in cancer and PAH, recent research relating miRNAs to pulmonary pathogenesis present a crucial need for more comprehensive studies about miRNAs' importance in PAH.

In the January 2013 edition of *Nature Medicine*, Kim et al. augmented the understanding of miRNAs role with exciting findings on miRNA-dependent association between apelin (APLN) and fibroblast growth factor 2 (FGF2) in PAH. Kim et al. studied angiogenic growth factors in normal PAECs with APLN knockdown and APLN and FGF2 mRNA levels in normal and PAH-PAECs (Kim et al., 2013). From these vital studies, the researchers revealed that a disruption of APLN signaling in PAECs results in increased expression of FGF2 and its receptor, FGFR1. Out of the 14 different types of miRNAs analyzed, Kim et al.'s critical experiment demonstrated that miR-424 and miR-503 were the most regulated microRNAs by APLN. The study suggested that transcription of miR-424 and miR-503, instead of their post transcriptional phase, is regulated by APLN signaling, since APLN knockdown caused downregulation of both pri and mature miR-424 and miR-503. The researchers studied the effects of miR-424 and miR-503 overexpression or knockdown on FGF2 and FGFR1 expression in normal and PAH-PAECs. Consequently, the experiment concluded that miR-424 and miR-503 regulate FGF2 and FGFR1, which are the key factors in endothelial FGF signaling process. The research concluded that the downregulation of APLN, miR-424 and miR-503 in PAH-PAECs increases FGF2 and FGFR1 expression and causes hyperproliferation of PAECs and PASMCs. To further investigate these miRNAs' role in the recovery of PAH-PAECs, the work demonstrated that miR-424 and miR-503 overexpression could inhibit the proliferation of PAECs in both normal and PAH samples by arresting the cell cycle at G0/GI stage. Interestingly, Kim et al.'s study results suggested that these miRNAs could affect proliferation of PASMCs in a paracrine manner. Most importantly, the experiment with rat models further validated the signaling axis between miR-424, miR-502, FGF2, and FGFR1. By intranasal administration of GFP-expressing control lentivirus or lentivirus expressing miR-424, miR-503, and GFP (424/503-GFP) to the rat models, the researchers found a reduction in right ventricular systolic pressure, fewer proliferating cellular nuclear antigens (PCNA), and a decreased expression of FGF2 and FGFR1 in 424/503-GFP treatment models. Therefore, the study concluded that the restoration of miR-424 and miR-503 inhibits FGF2 and FGFR1 expression and alleviates PAH pathogenesis.

With the use of experimental models of rodents and human alveolar-endothelial and smooth muscle cells at different stages of their study, Kim et al. have provided an extraordinary sense of optimism in the scientific community's search for more insights in pulmonary pathogenesis. However, Kim et al.'s study of the APLN-FGF link mediated by miRNAs compared only 14 miRNAs, which limited the study to only those miRNAs, even though a large number of miRNAs have been identified thus far. Further, the study results based on mmu-miR-322 and rno-miR-322, which are the mouse and rat homologs of has-miR-424, respectively, might provide an initial assessment; however, the application of the study results in humans with PAH truly depends on how comparative those homologs are to their corresponding miRNA within the human body. These study results can be further strengthened by clinical experiments in humans with PAH. Additionally, the plurality of miRNAs' targets, in addition to FGFR2, can warrant a more comprehensive knowledge of the molecular and cellular pathways. Despite the limitations, Kim et al. provided eloquent data that permits a rejuvenating guide for scientific studies to examine whether miRNAs can be used as biomarkers versus regulators of pulmonary pathophysiology. Hopefully, Kim et al.'s findings and other miRNA related studies will provide a foundation for exploration of therapeutic models for finding a cure for PAH.
